# Survival disparities in Australia: an analysis of patterns of care and comorbidities among indigenous and non-indigenous cancer patients

**DOI:** 10.1186/1471-2407-14-517

**Published:** 2014-07-18

**Authors:** Suzanne P Moore, Adèle C Green, Freddie Bray, Gail Garvey, Michael Coory, Jennifer Martin, Patricia C Valery

**Affiliations:** 1 Menzies School of Health Research, 147 Wharf St, Spring Hill, Brisbane 4000, Australia; 2 Cancer and Population Studies Group, Queensland Institute of Medical Research, 300 Herston Rd, Herston, 4006 Brisbane, Australia; 3 University of Manchester, Manchester Academic Health Science Centre, Manchester, UK; 4 International Agency for Research on Cancer, 150 Cours Albert Thomas, 69372 Lyon, France; 5 Murdoch Children’s Research Institute, Melbourne, Victoria, Royal Children’s Hospital, Flemington Road, Parkville, Melbourne 3052, Australia; 6 School of Medicine University of Queensland Translational Research Institute, 37 Kent ST, Woolloongabba 4071, Melbourne, Australia; 7 Monash University (Adjunct), Melbourne, Australia

**Keywords:** Indigenous, Cancer, Diabetes, Comorbidity, Disparity, Cancer stage, Survival, Queensland

## Abstract

**Background:**

Indigenous Australians have lower overall cancer survival which has not yet been fully explained. To address this knowledge deficit, we investigated the associations between comorbidities, cancer treatment and survival in Indigenous and non-Indigenous people in Queensland, Australia.

**Methods:**

A cohort study of 956 Indigenous and 869 non-Indigenous patients diagnosed with cancer during 1998–2004, frequency-matched on age, sex, remoteness of residence and cancer type, and treated in Queensland public hospitals. Survival after cancer diagnosis, and effect of stage, treatment, and comorbidities on survival were examined using Cox proportional hazard models.

**Results:**

Overall Indigenous people had more advanced cancer stage (p = 0.03), more comorbidities (p < 0.001), and received less cancer treatment (77% vs. 86%, p = 0.001). Among patients without comorbidities and social disadvantage, there was a lower uptake of treatment among Indigenous patients compared to non-Indigenous patients. For those who received treatment, time to commencement, duration and dose of treatment were comparable. Unadjusted cancer survival (HR = 1.30, 95% CI 1.15-1.48) and non-cancer survival (HR = 2.39, 95% CI 1.57-3.63) were lower in the Indigenous relative to non-Indigenous patients over the follow-up period. When adjusted for clinical factors, there was no difference in cancer-specific survival between the groups (HR = 1.10, 95% CI 0.96-1.27). One-year survival was lower for Indigenous people for all-causes of death (adjusted HR = 1.33, 95% CI 1.12-1.83).

**Conclusion:**

In this study, Indigenous Australians received less cancer treatment, had more comorbidities and had more advanced cancer stage at diagnosis, factors which contribute to poorer cancer survival. Moreover, for patients with a more favourable distribution of such prognostic factors, Indigenous patients received less treatment overall relative to non-Indigenous patients. Personalised cancer care, which addresses the clinical, social and overall health requirements of Indigenous patients, may improve their cancer outcomes.

## Background

In Australia, cancer is the second leading cause of death among Indigenous people, who continue to experience a significantly lower life-expectancy than the population as a whole. The observation of lower cancer survival among Indigenous compared to non-Indigenous Australians is well established [1-3] but not as yet fully explained. Factors contributing to this survival disparity include later stage of cancer at diagnosis, reduced uptake of or access to treatment, high rates of case-fatal cancers and co-morbidities, and language barriers [1,4,5]. A previous study in the state of Queensland found that Indigenous people with cancer were more likely to have comorbidities, to receive less treatment, and to experience worse survival than non-Indigenous counterparts [6].

We replicated this earlier matched-cohort study design where we compared Indigenous and non-Indigenous people with cancer, this time including a larger cohort, collecting more comprehensive and detailed information on cancer treatment (e.g. timing to, type and amount of treatment) and comorbidities. Here we examine the associations between comorbidities, cancer treatment and survival among Indigenous and non-Indigenous cancer patients in Queensland, the state with the second-largest Indigenous population in Australia.

## Methods

Methods for this comparative study of Indigenous and non-Indigenous cancer patients are similar to those described previously [6,7]. Briefly, all Indigenous adults residing in the state of Queensland and diagnosed with cancer during 1998–2004, identified through the population-based Queensland Cancer Registry (QCR), were eligible for inclusion. An equal number of non-Indigenous cases were randomly identified from the Registry (frequency-matched for age, sex, remoteness and cancer type). All Australian residents have access to free public-health care, including cancer treatment, and those with private insurance, or the means to pay, can also access treatment in the private sector [6]. We restricted our cohort to those who received the majority of treatment in a Queensland public hospital, as about 98% of Indigenous patients receive care in the public sector [8]. Patients primarily treated elsewhere, those with missing health records, or those treated in a hospital where regulatory approval was not forthcoming, were excluded.

Clinical data (diagnostic details, cancer treatment, cancer stage, and presence of comorbidities), were abstracted from medical records, as this information was not available from the QCR. Where records were insufficiently detailed, further data were extracted from secondary public hospitals’ records, to ensure that cancer treatment and other clinical data were as complete as possible. In general, details of surgery, chemotherapy or radiotherapy treatment that occurred outside the public hospital system were documented in the public health records, through treating clinician’s letters. Details on all comorbidities recorded in the patients’ medical records were extracted; comorbidities that fitted with the Charlson Comorbidity Index Score (CCI) were included in the analysis. A modified CCI score (referred to here as ‘comorbidity score’) was assigned based on severity and number of comorbid conditions [9] and were grouped as: 0 (No known comorbidity), 1, 2+. Modification of the original CCI was necessary where data on the severity of renal or liver disease were not collected; these were classified as 2. Remoteness (rurality of residence) was determined using the Accessibility/Remoteness Index of Australia [10] with groups ranging from 1 ‘highly accessible’ and 5 ‘very remote’. For multivariate analysis, the categories were aggregated to three groups; 1 (highly accessible and accessible), 2 (moderately accessible) and 3 (remote and very remote). The Socio-Economic Index For Areas (SEIFA) was used to classify place of residence into quintiles ranging from 1 ‘most disadvantaged’ to 5 ‘most advantaged’ [11].

Cancer stage scores such as Tumour Nodes Metastasis (TNM), Dukes and American Joint Committee on Cancer (AJCC) [12] staging were converted to localised/regional/distant spread. Treatment type (surgery, radiotherapy and chemotherapy), intention (any intent, curative intent or intention unknown), start date, duration, and quantity (e.g. number of Gray (Gy), number of chemotherapy cycles) were recorded. Date and cause of death were obtained from the Australian National Death Index. All cases were followed-up with respect to their vital status until Dec 31, 2006.

Ethical approval was obtained from the Queensland Health Department, health districts where data collection took place, and the Queensland Institute of Medical Research.

### Statistical methods

Pearson’s Chi-squared analysis or Fisher’s Exact test were used for categorical data (proportions), t-test for normally-distributed data (means), and non-parametric tests (Kruskal-Wallis Test) for non-normally-distributed data (medians). We estimated the relative risk of no treatment associated with comorbidity by calculating the odds ratios (OR) and 95% confidence interval (CI) using multivariate logistic regression analysis. Time to death was assessed using Kaplan-Meier survival curves. The curves were compared with the log-rank test statistic. Cox proportional hazard modelling was used to calculate hazard ratios (HRs) with associated 95% CI to assess the differences between Indigenous and non-Indigenous cases with respect to cancer survival (all-cause, cancer-specific and non-cancer), after adjustment for cancer stage, comorbidities, socioeconomic status and treatment [13]. All cases were followed-up from diagnosis until death or Dec 31, 2006, whichever came sooner; cases who were still alive at December 31, 2006 were censored at that date.

## Results

The study included 956 Indigenous and 869 non-Indigenous patients (368 cases were excluded). Clinical data were abstracted from records at 44 hospitals. Matching resulted in similar distribution of age, sex and site of cancer diagnosis between the two groups. However, perfect matching for remoteness was not possible as fewer non-Indigenous people with cancer lived in the most remote locations at the time of diagnosis (Table 1). Indigenous people were more likely to be socially disadvantaged than their non-Indigenous counterparts (p < 0.001) (Table 1).

**Table 1:** **Demographic and clinical characteristics**, **and patterns of care among Indigenous and non**-**indigenous cancer patients**, **Queensland**, **Australia 1998**–**2004**

	**Indigenous (n** **=** **956) N (%)**	**Non-Indigenous (n** **=** **869) N (%)**	**P-value**
**Age**			
18- 39 years	108 (11)	91 (11)	0.852
40 – 59 years	421 (44)	387 (45)	
60 + years	427 (45)	391 (45)	
**Sex**			
male	436 (46)	393 (45)	0.870
female	520 (54)	476 (55)	
**Area of remoteness index**			
Highly accessible/Accessible	329 (34)	369 (43)	<0.001
Moderately accessible	370 (39)	325 (37)	
Remote/Highly remote	257 (27)	175 (20)	
**Socioeconomic status** (**SEIFA**)			
1 Most disadvantaged	354 (37)	215 (25)	<0.001
2 Disadvantaged	238 (25)	261 (30)	
3 Intermediate advantage	213 (22)	219 (25)	
4 Advantaged	111 (12)	136 (16)	
5 Most advantaged	39 (4)	36 (4)	
Cancer stage at diagnosis*			
Localised cancer	316 (38)	336 (45)	0.030
Regional Spread	240 (29)	197 (26)	
Distant metastasis	275 (33)	221 (29)	
Unknown or not applicable	125	115	
Comorbidity score**			
0	481 (50)	601 (69)	<0.001
1	256 (27)	143 (16)	
2+	219 (23)	125 (14)	
**Diabetes**			
No	669 (70)	785 (90)	<0.001
Yes	287 (30)	84 (10)	
**HbA1C levels recorded**	(N = 113)	(N = 18)	
Equal to or less than 6.5%	36 (32)	7 (39)	0.485
Greater than 6.5%	77 (68)	11 (61)	
**Any cancer treatment**			
Given treatment	716 (75)	745 (86)	<0.001
No treatment or treatment unknown	240 (25)	124 (14)	
**Curative cancer treatment**^**£**^	**N** **=** **556**	**N** **=** **533**	
Given treatment	400(72)	417 (78)	0.030
No treatment or treatment unknown	153 (28)	116 (22)	
**Surgery**			
Surgery	494 (52)	549 (64)	<0.001
No surgery or treatment unknown	462 (48)	320 (36)	
**Chemotherapy**			
Chemotherapy	269 (28)	321 (37)	<0.001
No chemotherapy or treatment unknown	687 (72)	548 (63)	
**Chemotherapy completed^**	**N** **=** **160**	**N** **=** **190**	
Completed	122 (76)	152 (80)	0.530
Not completed or treatment unknown	38 (24)	38 (20)	
**Radiotherapy**			
Radiotherapy	325 (34)	359 (41)	0.001
No Radiotherapy or not sure	631 (66)	510 (59)	
**Age**			
18- 39 years	108 (11)	91 (11)	0.852
40 – 59 years	421 (44)	387 (45)	
60 + years	427 (45)	391 (45)	
**Sex**			
male	436 (46)	393 (45)	0.870
female	520 (54)	476 (55)	
**Area of remoteness index**			
Highly accessible/Accessible	329 (34)	369 (43)	<0.001
Moderately accessible	370 (39)	325 (37)	
Remote/Highly remote	257 (27)	175 (20)	
**Socioeconomic status (SEIFA)**			
1 Most disadvantaged	354 (37)	215 (25)	<0.001
2 Disadvantaged	238 (25)	261 (30)	
3 Intermediate advantage	213 (22)	219 (25)	
4 Advantaged	111 (12)	136 (16)	
5 Most advantaged	39 (4)	36 (4)	
**Cancer site and IDC code**^**#**^			
Bronchus and lung (C33- C34)	187 (20)	173 (20)	0.9
Breast (C56)	111 (12)	107 (12)	
Colorectal and small intestine (C17-20)	87 (9)	89 (10)	
Head and neck (C00- C14)	83 (9)	84 (10)	
Lymphoma and leukaemia (C42 & C77/ M 01- M10)	61 (6)	55 (6)	
Cervix (C53)	56 (6)	49 (6)	
Liver (C22)	48 (5)	30 (4)	
Other cancers	41 (4)	32 (4)	
Renal tract (C64- C67)	40 (4)	46 (5)	
Corpus uteri (C54)	38 (4)	36 (4)	
Oesophagus (C15)	31 (3)	21 (2)	
Other cancers	47 (5)	35 (4)	
Ovary (C56)	29 (3)	29 (3)	
Unknown primary (C80)	29 (3)	17 (2)	
Stomach (16)	27 (3)	23 (3)	
Prostate (C61)	25 (3)	17 (2)	
Pancreas (C25)	22 (2)	25 (3)	
Thyroid (C73)	21 (2)	14 (2)	
Central Nervous System (C71)	13 (1)	12 (1)	
Melanoma (C44 or M12))	7 (1)	10 (1)	
Cancer stage at diagnosis*			
Localised cancer	316 (38)	336 (45)	0.030
Regional Spread	240 (29)	197 (26)	
Distant metastasis	275 (33)	221 (29)	
Unknown or not applicable	125	115	
Comorbidity score**			
0	481 (50)	601 (69)	<0.001
1	256 (27)	143 (16)	
2+	219 (23)	125 (14)	
**Diabetes**			
No	669 (70)	785 (90)	<0.001
Yes	287 (30)	84 (10)	
**HbA1C levels recorded**	(N = 113)	(N = 18)	
Equal to or less than 6.5%	36 (32)	7 (39)	0.485
Greater than 6.5%	77 (68)	11 (61)	
**Any cancer treatment**			
Given treatment	716 (75)	745 (86)	<0.001
No treatment or treatment unknown	240 (25)	124 (14)	
**Curative cancer treatment**^**£**^	**N** **=** **556**	**N** **=** **533**	
Given treatment	400 (72)	417 (78)	0.030
No treatment or treatment unknown	153 (28)	116 (22)	
**Surgery**			
Surgery	494 (52)	549 (64)	<0.001
No surgery or treatment unknown	462 (48)	320 (36)	
**Chemotherapy**			
Chemotherapy	269 (28)	321 (37)	<0.001
No chemotherapy or treatment unknown	687 (72)	548 (63)	
**Chemotherapy completed^**	**N** **=** **160**	**N** **=** **190**	
Completed	122 (76)	152 (80)	0.530
Not completed or treatment unknown	38 (24)	38 (20)	
**Radiotherapy**			
Radiotherapy	325 (34)	359 (41)	0.001
No Radiotherapy or treatment unknown	631 (66)	510 (59)	

^#^International Classification of Diseases 10th Revision (ICD-10-AM) code.

*Cases with missing data not included in analysis but numbers presented for completeness.

**Charlson Comorbidity Index: scores 2–5 and 5+ were grouped.

^£^Includes cases with localised and regional stage disease only.

^Includes only cases with non-metastatic disease who received chemotherapy.

Median time from presentation to diagnosis was 17 days for both groups. Cancer stage was not recorded in the clinical notes in approximately 10% of patients in both groups; a further 3% of cases in each group were diagnosed with cancers for which a stage was not routinely recorded (e.g. leukaemias, lymphomas). With these cases excluded from analysis, fewer Indigenous people were diagnosed with local disease compared to non-Indigenous people (38% vs. 45%, p = 0.03). There was no difference in cancer stage among men (p = 0.65) but fewer Indigenous women had localised cancer (41% vs. 51%), and 59% had regional spread or distant metastasis compared to 48% for non-Indigenous women (p = 0.007). Analysis, stratified by remoteness, showed no difference in stage between Indigenous and non-Indigenous people from the same regions (data not shown).

Indigenous patients were more likely to have comorbidities than their counterparts. They were significantly less likely to have a zero comorbidity score and more likely to have a score of 2 or greater (p < 0.001) (Table 1). HbA1c, a measure of diabetes control [14], was included if tested in the year prior to diagnosis or within 3 months of diagnosis. Measurements were available for 40% of Indigenous and 21% of non-Indigenous patients with diabetes, with no difference in the proportion of cases with HbA1c over 6.5%. Median measurements were 7.3% and 6.9% respectively, and were similar regardless of sex or remoteness.

**Figure 1:**
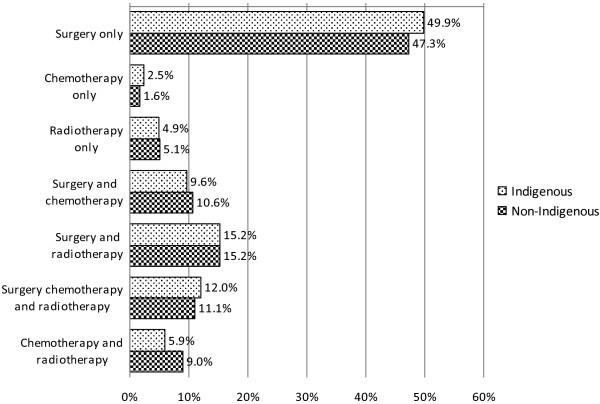
**Comparison of mode of treatment received by Indigenous and non**-**Indigenous cancer patients (p** **=** **0.910).** *metastatic cases excluded.

**Figure 2:**
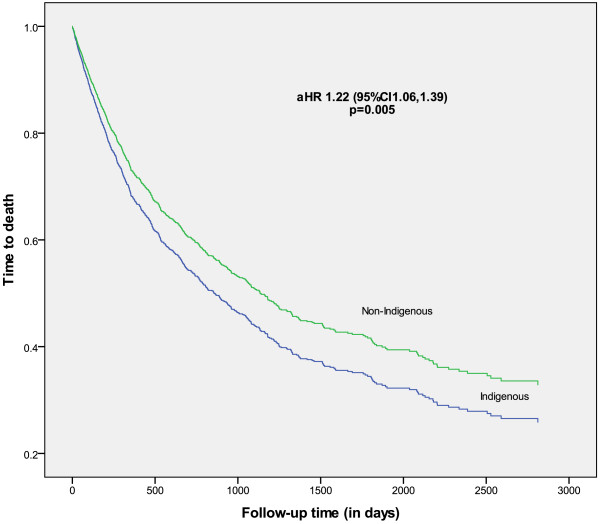
**Time from diagnosis to all-cause death for Indigenous and non-Indigenous people with cancer, adjusted for age, sex, cancer site, place of residence (ARIA), socioeconomic (SEIFA), comorbidity score (CCI), stage at diagnosis, treatment (follow-up until 31**^**st **^**Dec 2006).**

Indigenous cancer patients received less of any treatment (75% vs. 86%, p < 0.001) and for those with non-metastatic disease, received less treatment with curative intent (Table 1). Irrespective of sex, stage, remoteness, or socioeconomic status, Indigenous people received less treatment (data not shown). Since matching was imperfect for place of residence (remoteness), in a sub-group analysis we excluded people from the most remote regions: Indigenous people from rural areas (77% vs. 89%, p < 0.001) and those from more urban areas (83% vs. 90%, p = 0.01) received less treatment (any, surgery, chemotherapy or radiotherapy). Among patients who received treatment, modes of curative cancer treatment were similar (Figure 1). Indigenous people (n = 44) who concurrently lived in cities, had no known comorbidity and no metastatic cancer, and were not socially disadvantaged, took up or received less treatment than non-Indigenous counterparts (n = 73) (82% vs. 95%, p = 0.05) (Figure 2). Patients with diabetes and HbA1c measurements greater than 6.5% received similar rates of treatment (n = 62 (81%) vs. n = 9 (82%); p = 0.93) as those without diabetes.

Being Indigenous increased the odds of not receiving any cancer treatment and curative chemotherapy (Table 1). Stratified analyses showed that among cases who had no comorbidities recorded in the medical chart, being Indigenous increased the likelihood of not taking up or receiving any cancer treatment and curative surgery; that was also true among cases who had a comorbidity score of one.

The median time from date of diagnosis to the first cancer treatment was 14 days for Indigenous (n = 698) and 12 days for non-Indigenous people (n = 734; p = 0.20). There was no significant difference in the median number of days to first surgical treatment, chemotherapy or radiotherapy between the two groups. For cases without metastatic disease, there was no difference between Indigenous and non-Indigenous people in the duration or quantity of radiotherapy (52.5 Gy respectively) or number of cycles of chemotherapy administered (p = 0.93). Also, there was no difference in the proportion of people with non-metastatic cancer who completed chemotherapy (76% of Indigenous patients and 80% of non-Indigenous patients completed chemotherapy regimen; p = 0.53) (Table 2).

**Table 2:** **Odds of receiving treatment in Indigenous compared to non**-**Indigenous** (**referent group**) **cancer patients**: **all cases and stratified by comorbidity score**^

		Adjusted OR* (95% CI)
**Any treatment**		**0.46 (0.41, 0.63)**
Stratified by:	comorbidity score 0	**0.36 (0.23, 0.57)**
	comorbidity score 1	**0.43 (0.23, 0.81)**
	comorbidity score 2+	0.73 (0.39, 1.34)
**Curative treatment** #		0.74 (0.56, 1.00)
Stratified by:	comorbidity score 0	0.76 (0.46, 1.03)
	comorbidity score 1	0.55 (0.28, 1.09)
	comorbidity score 2+	1.29 (0.65, 2.55)
**Curative surgery** #		0.82 (0.62,1.07)
Stratified by:	comorbidity score 0	**0.68 (0.48, 0.97)**
	comorbidity score 1	0.86 (0.46, 1.60)
	comorbidity score 2+	1.49 (0.74, 2.91)
**Curative chemotherapy** #		**0.72 (0.53, 098)**
Stratified by:	comorbidity score 0	0.79 (0.55, 1.13)
	comorbidity score 1	0.62 (0.30, 1.28)
	comorbidity score 2+	0.36 (0.12, 1.07)
**Curative radiotherapy** #		0.82 (0.63, 1.07)
Stratified by	comorbidity score 0	0.88 (0.64), 1.23)
	comorbidity score 1	0.56 (0.29, 1.08)
	comorbidity score 2+	0.66 (0.31, 1.42)

*Adjusted for age, sex, remoteness, socioeconomic status, stage, comorbidity score.

# metastatic cases excluded.

^Comorbidity Score: 0 (no known comorbidity), 1 (Charlson index of 1) or 2+ (Charlson index of 2 or more).

**Figure 3:**
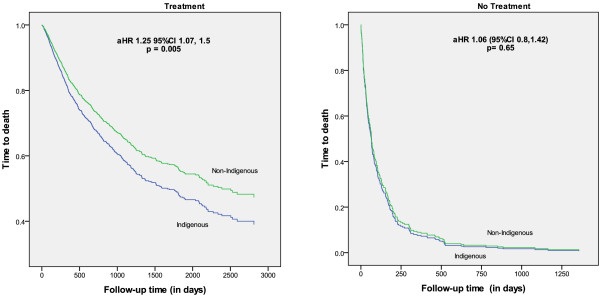
**Time from diagnosis to all-cause death for Indigenous and non**-**Indigenous people with respect to treatment**, **adjusted for age, sex, cancer site, place of residence (ARIA), socioeconomic (SEIFA), comorbidity score (CCI), stage at diagnosis, treatment (Follow-up until 31**^**st **^**Dec 2006).**

Compared to non-Indigenous patients, the unadjusted difference in overall survival was 37% worse for Indigenous patients over the study period (Figure 3). Cancer-specific survival and non-cancer survival were also significantly lower (Table 3). The differential cancer-specific survival did not remain significant after adjusting for demographic and clinical factors, but persisted for non-cancer survival. Overall survival was 60% lower for Indigenous people in the first year after diagnosis, but not significantly different in subsequent years. The survival differential persisted after adjustment for demographic factors, stage, comorbidities, and treatment reduced the hazard ratio for the first year (aHR = 1.33 95% CI 1.12-1.83), but not for subsequent years. Overall, survival for Indigenous people who received treatment was lower than for non-indigenous counterparts (aHR = 1.26 95% CI 1.07-1.5), whereas survival was similar for those who did not receive treatment (Figure 3).

**Table 3:** **Proportional hazard ratios**, **using Cox regression models**, **of time to death for all cancer diagnosed between 1998 and 2004**, **for Indigenous compared to non**-**Indigenous people in Queensland**, **Australia**

		**HR**	**(95% CI)**	aHR*	**(95% CI)**
All cause death		1.37	(1.22, 1.55 )	1.22	(1.06-1.39)
Non-cancer death		2.39	(1.57, 3.63)	1.95	(1.17, 3.24)
Cancer death		1.30	(1.15, 1.48 )	1.10	(0.96, 1.27)
Time to death (all causes) by years:	0 – 1 yr	1.60	(1.37, 1.87)	1.33	(1.12, 1.83)
	1 – 2y	0.98	(0.73, 1.33)	1.18	(0.85, 1.65)
	2- 3 yr	0.98	(0.76, 1.26)	0.98	(0.77, 1.26)
	3- 4 yr	0.97	(0.74, 1.30)	1.03	(0.79 1.36)
	4- 5 yr	1.05	(0.76, 1.45)	1.02	(0.72, 1.45)

*Adjusted for: Sex, Age, Comorbidity Score (Modified Charlson Comorbidity Index): 0, 1, 2+; Accessibility/Remoteness Index of Australia 1 = Highly accessible/Accessible 2 = Moderately accessible 3 = Remote/Highly remote; SEIFA (Socioeconomic Index for Areas): 1 = Most disadvantaged, 2 = Intermediate advantage, 3 = Most advantaged; Stage: 1 = Localised, 2 = Regional Spread, 3 = Metastatic Disease, 9 = Not sure; Any treatment.

## Discussion

Compared with non-Indigenous patients resident in Queensland, Australia, Indigenous people were diagnosed with more advanced cancer, had greater comorbidity, received less cancer treatment and were 30% more likely to die of their cancer; findings similar to those reported previously [5,6,15]. Concomitant disease is reported to influence cancer treatment choices (dose, duration, modality), cause complications, and impede survival [9,16-18]. Excess comorbidities have been shown to contribute to lower curative treatment rates and poorer cancer survival for New Zealand Māori [18,19], and contributed to the survival disparity among Indigenous people with lung cancer in Queensland [20]. Indigenous people with lung cancer and diabetes had reportedly 40% worse survival than Indigenous people without diabetes, whereas the presence of diabetes was unrelated to survival in non-Indigenous people [21].

In this study, stratified analysis showed that among cases without comorbidity, being Indigenous was associated with not receiving any cancer treatment, including curative surgery. This suggests that factors other than or in conjunction with comorbidity status may play a role. Of particular concern are the reasons why Indigenous people without comorbidities, who lived in an urban setting where cancer services are easily accessible, were not socially disadvantaged, and had localised cancer at diagnosis, did not have treatment. Better identification of reasons for treatment disparities in this group may enable cancer care providers to increase treatment uptake and in turn improve overall survival. Despite thorough review, we found that reasons for not receiving treatment were not routinely documented in the medical charts. We thus recommend that the views of patients and health services personnel are sought and documented to elicit and analyse barriers to treatment for Indigenous people. Our study was unable to capture sufficient information regarding comorbidities not included in the Charslon Comorbidity Index (e.g. mental illness, alcoholism), factors which might preclude cancer treatment.

Our findings regarding treatment parallel reports from a number of international [22-24] and local [6,25-27] studies. We found that irrespective of remoteness or socioeconomic advantage, Indigenous people received less treatment than non-Indigenous counterparts (including Indigenous people who were most advantaged). Factors such as miscommunication and social and cultural differences between patients and health care providers [28,29], poor understanding of medical advice [30], inherent racism in the health system [31], lack of transport [32], geographic isolation [33], and lack of Indigenous support persons [34], have been cited as some of the reasons for treatment disparity. We found no delay for patients receiving treatment in public hospitals, in contrast to earlier studies which reported a significant difference in time to surgery [6] and delays in treatment [18,35]. We also report that the presence of diabetes or elevated HbA1C did not influence treatment uptake and that chemotherapy completion rates for those receiving treatment for non-metastatic cancer, were similar for both groups.

A greater proportion of Indigenous people in our cohort had died before the end of the follow-up period, and they were twice as likely to have died from a non-cancer death. These results are consistent with the poor cancer survival and higher mortality that have been reported for Indigenous people with cancer elsewhere [36-39]. Unadjusted analysis indicated a lower survival observed among Indigenous compared to non-Indigenous people (all causes, non-cancer and cancer deaths) and the survival differential for cancer death remained apparent when adjusted for confounding factors (age, sex, remoteness, stage, socioeconomic status and comorbidities). When further adjusted for diabetes and/or treatment, the differential reduced to borderline significance, suggesting these factors may be associated with lower survival. Despite this, we found that overall Indigenous people who had treatment fared worse than non-indigenous people who had treatment, suggesting either the type or quality of treatment, or other non-treatment factors at play in overall treatment outcomes.

Survival was worse for Indigenous people in the first year after diagnosis. After adjustment for stage and comorbidities, the survival differential persisted in the first year but, when adjusted for treatment alone, this difference was no longer apparent. Treatment differences and, to a lesser extent, stage and comorbidities, may be important contributing factors for the poorer outcomes immediately after cancer diagnosis among Indigenous people. Our findings are similar to a recent Queensland report which included patients treated at public and private hospitals [2]; Indigenous people were 1.5 times more likely to die in the first year of diagnosis than non-Indigenous people, but with excess mortality limited to the first two years of follow-up after diagnosis. Although that study was much larger than the present one, it was limited by a lack of information on cancer stage, comorbidities and cancer treatment. Our results, adjusted for treatment, stage and comorbidities, suggest that differences in treatment between the two groups are mainly responsible for the survival differential in the first year after a cancer diagnosis.

Misidentification of Indigenous status in Queensland public hospitals is known to be around 12% [40]. As Indigenous status was checked in the medical record review and discrepancies investigated where possible, we are confident that misclassification in the study sample is minimal. Matching resulted in little differences in the distribution of age, sex or cancer type between the two groups, however, as perfect matching was not possible, it resulted in the inclusion of a higher proportion of Indigenous people who lived very remotely and were severely disadvantaged. Of note, 27% of Indigenous people in this study were from the more remote regions, whereas 22% of the Indigenous population resident in Queensland live in these regions [41]. This minimizes the concern that Indigenous people from remote regions, who potentially have inadequate access to the health care system, might have been underrepresented in this study. The chief strength in utilizing data from the National Death Index (NDI) for vital status is the virtually complete population coverage. Standardized and consistent data collection by the NDI ensures that the death data are of high quality (93.7% sensitivity and 100% specificity for the identification of deaths) [42]. Nevertheless, if a few cases were missed due to reporting delays to the NDI, they are not likely to be differentially biased by Indigenous status.

## Conclusions

This comprehensive investigation of the patterns of care of Indigenous people with cancer, confirmed that Indigenous people had more advanced stage at diagnosis, more comorbidities, received less treatment, and had poorer survival than their non-Indigenous counterparts. In addition, we found that survival disparity in the first year after diagnosis was likely to be related to lack of cancer treatment. However, Indigenous people receiving cancer treatment had poorer outcomes overall, despite comparable time to commencement, duration, and amount of treatment received. We also identified a subset of untreated patients who might reasonably be considered candidates for cancer treatment. A greater understanding of the interplay between overall health, demographic features and cancer, as well as Indigenous people’s awareness of cancer and cancer treatment, is therefore required. Indigenous cancer patients have particular needs that should be considered when planning cancer care, particularly during the first year after diagnosis.

## Competing interests

The authors declare that they have no competing interests.

## Authors' contributions

S P Moore a participated in the conception, design, analyses of the data, interpretation of results, writing and editing the manuscript. A Green participated in the conception, design, analyses of the data, interpretation of results and editing the manuscript. F Bray assisted in editing the manuscript. G Garvey participated in the interpretation of results and editing the manuscript. J Martin participated in the interpretation of results and editing the manuscript. M Coory participated in the conception, design, analyses of the data, interpretation of results. P Valery participated in the conception, design, analyses of the data, interpretation of results and editing the manuscript. We confirm that all authors have seen and approved its final version.

## Pre-publication history

The pre-publication history for this paper can be accessed here:

http://www.biomedcentral.com/1471-2407/14/517/prepub
